# Lie group analysis of magnetohydrodynamic tangent hyperbolic fluid flow towards a stretching sheet with slip conditions

**DOI:** 10.1016/j.heliyon.2017.e00443

**Published:** 2017-11-03

**Authors:** Zakir Ullah, Gul Zaman

**Affiliations:** Department of Mathematics, University of Malakand, Chakdara, Dir (Lower), Khyber Pakhtunkhawa, Pakistan

**Keywords:** Applied mathematics, Mechanics

## Abstract

In this paper, we studied MHD two dimensional flow of an incompressible tangent hyperbolic fluid flow and heat transfer towards a stretching sheet with velocity and thermal slip. Lie group analysis is used to develop new similarity transformation, using these similarity transformation the governing nonlinear partial differential equation are reduced into a system of coupled nonlinear ordinary differential equation. The obtained system is solved numerically by applying shooting method. Effects of pertinent parameters on the velocity and temperature profiles, skin friction, local Nusselt number are graphically presented and discussed. Comparison between the present and previous results are shown in special cases.

## 1 Introduction

The study of the non-Newtonian fluids has gained a considerable interest in the last few decades due to its numerous applications in different fields of science and technology. Honey, gels, paints, blood, printer inks, lubricant containing polymer additives, cosmetics and toiletries are some of the common examples of non-Newtonian fluids. Tangent hyperbolic fluid model is a particular type of non-Newtonian fluid which is used extensively for different laboratory experiments. Akber et al. [1] studied two dimensional tangent hyperbolic fluid toward a stretching sheet with a magnetic field. Friedman et al. [2] studied the hyperbolic tangent fluid model for the large-scale magneto-rheological fluid damper coils. Nadeem and Akram [3] analyzed peristaltic transport of a hyperbolic tangent fluid model in a symmetric channel. Naseer et al. [4] analyzed the boundary layer flow and heat transfer of a hyperbolic tangent fluid flowing over a vertical exponentially stretching cylinder in its axial direction. Jyothi et al. [5] investigated the peristaltic transport of a hyperbolic tangent fluid through a porous medium in a two-dimensional symmetric inclined channel.

The flow due to stretching sheet has attracted a considerable attention of the researchers. Sakiadis [6] studied laminar bounded-layer behavior on a moving continuous flat surface and has obtained numerical solation for the boundary-layer equations. Crane [7] has extended the work for both liner and exponentially stretching sheet. The flow due to a stretching surface with partial slip has been study by Wang [8]. Nazarn et al. [9] investigated the unsteady two-dimensional stagnation point flow of the incompressible viscous fluid over a flat deformable sheet. Hayat et al. [10] studied the magnetohydrodynamic (MHD) flow and heat transfer characteristics for the boundary layer flow over a permeable stretching sheet. Zaimi and Ishak [11] analyzed the effects of partial slip on stagnation-point flow and heat transfer due to a stretching vertical sheet. The influence of buoyancy along with viscous dissipation on the convective transport in a boundary layer region in both aiding and opposing flow situation is discussed by Partha et al. [12]. Ishak et al. [13] studied heat transfer over a stretching surface with uniform or variable heat flux in micropylar fluid. Nadeem and Hussain [14] analyzed HAM solation for boundary layer flow in the region of the stagnation point toward a stretching sheet.

Sophius Lie [15] developed a classical method known as Lie group analysis to find point transformation that map a given differential equation to itself. This method unifies almost all known exact integration techniques for both ordinary and partial equation [16]. Lie group analysis used to find the similarity reduction of the non-linear differential equations. In such analysis, one reduces the number of variable governing the partial differential equations. This reduction of variables changes the system of partial differential equations to self similar system of the ordinary differential equations. Lie group analysis has been used to analyzed convective phenomena under various flow configurations that arise in various branch of science and engineering [17]. Avramenko et al. [18] used Lie group technique to investigate the symmetric properties of turbulent boundary-layer flows. Using a Lie group analysis, Kuznetsov et al. [19] theoretically investigated a falling bioconvection plume in a deep chamber filled with a fluid saturated porous medium. On the boundary-layer flow, Hamad et al. [20] used Lie group technique to study the effect of thermal radiation and convective surface boundary condition. This analysis has been used to study mixed convective flow in the presence of mass transfer [21]. With the temperature dependent thermal conductivity, Aziz et al. [22] studied the variable reactive index and heat generation in MHD flow over an inclined radiating plate. Kandasamy et al. [23] analyzed a steady two-dimensional flow of an electrically conducting incompressible fluid over a vertical stretching sheet by using scaling group of transformations to do so. Rashidi et al. [24] found Lie group solution for free convective flow of a nanofluid past a chemically reacting horizontal plate in a porous media.

In this paper, we have included the work of Akber et al. [1] by considering heat transfer along with velocity and thermal slip for tangent hyperbolic fluid flow towards a stretching sheet. Lie group analysis is used to develop new similarity transformation, using these similarity transformation the governing nonlinear partial differential equation are reduced into a system of coupled nonlinear ordinary differential equation. The obtained system of coupled nonlinear ordinary differential equation subject to the boundary conditions is solved with the help of shooting method. The effects of various parameters on the velocity and temperature are presented graphically. The skin friction coefficient and heat transfer rates are discussed quantitatively.

## 2 Model

We consider a steady, two dimensional flow of an incompressible tangent hyperbolic fluid. We assume that the fluid is coincident with the plane y=0, such that the flow is limited to the region y>0. We further assume that the generation of the fluid is due to the linear stretching. As describe in [25] the governing equation of the fluid is given by

(1) $$\overset{¯}{\tau }=\left[\mu_{\infty }+\left(\mu_{0}+\mu_{\infty }\right)\tanh{\left(\Gamma \overset{¯}{\overset{˙}{\gamma }}\right)}^{n}\right]\overset{¯}{\overset{˙}{\gamma }}.$$

Here $\overset{¯}{\tau }$, $\mu_{0}$, $\mu_{\infty }$, Γ and *n* denote the extra stress tensor, the infinite shear rate viscosity, the zero shear rate viscosity, the time dependent material constant and the flow behavior index respectively. The term $\overset{¯}{\overset{˙}{\gamma }}$ is given by

(2) $$\overset{¯}{\overset{˙}{\gamma }}=\sqrt{\frac{1}{2}\Sigma_{i}\Sigma_{j}\overset{¯}{\overset{˙}{\gamma_{ij}}}\overset{¯}{\overset{˙}{\gamma_{ji}}}}=\sqrt{\frac{1}{2}\Pi },$$

with $\Pi =\frac{1}{2}tr{\left(gradV+(gradV^{T})\right)}^{2}$. As we are concerned with infinite shear rate viscosity problem, so in the following we restrict ourselves to the case $\mu_{\infty }=0$. The fluid under consideration that described shear thinning, so we must take $\Gamma \overset{¯}{\overset{˙}{\gamma }}<1$. Then Eq. (1) reduces to

(3) $$\begin{array}{c} \overset{¯}{\tau }=\mu_{0}\left[{\left(\Gamma \overset{¯}{\overset{˙}{\gamma }}\right)}^{n}\right]\overset{¯}{\overset{˙}{\gamma }}=\mu_{0}\left[{\left(1+\Gamma \overset{¯}{\overset{˙}{\gamma }}-1\right)}^{n}\right]\overset{¯}{\overset{˙}{\gamma }},\\ =\mu_{0}\left[1+n\left(\Gamma \overset{¯}{\overset{˙}{\gamma }}-1\right)\right]\overset{¯}{\overset{˙}{\gamma }}. \end{array}$$

The governing equations of the continuity, momentum and energy for the proposed model are

(4) $$\frac{\partial u}{\partial x}+\frac{\partial v}{\partial y}=0,$$

(5) $$u\frac{\partial u}{\partial x}+v\frac{\partial u}{\partial y}=\nu (1-n)\frac{\partial^{2}u}{\partial y^{2}}+\sqrt{2}\nu n\Gamma \left(\frac{\partial u}{\partial y}\right)\frac{\partial^{2}u}{\partial y^{2}}-\frac{\sigma B^{2}}{\rho }u,$$

(6) $$u\frac{\partial T}{\partial x}+v\frac{\partial T}{\partial y}=\frac{\kappa }{\rho c_{p}}\frac{\partial^{2}T}{\partial y^{2}}+\frac{Q_{0}}{\rho cp}(T-T_{\infty }).$$

Here, *u* and *v* are the velocity components in the *x*- and *y*-direction, respectively. The kinematic fluid viscosity and the fluid density are respectively expressed by the symbols *ν* and *ρ*. *σ* is the electrical conductivity of the fluid, the term *B* is denoted the applied uniform magnetic field. The temperature and the free stream temperature are respectively denoted by *T* and $T_{\infty }$. The specific heat, the thermal conductivity of the fluid and volumetric rate of the heat generation are respectively denoted by $c_{p}$, *κ* and $Q_{0}$.

The associative boundary conditions for the velocity components and temperature are given by

(7) $$u=ax+L\frac{\partial u}{\partial y},\;v=0,T=T_{w}+D_{1}\frac{\partial T}{\partial y},\;\text{at}\;y=0,$$

(8) $$u\to 0,\;T\to T_{\infty },\;\text{as}\;y\to \infty .$$

Here, *a* is the stretching rate, *L* is the velocity slip factor and $D_{1}(x)$ is the thermal slip factor. For $L=0=D_{1}(x)$ the no slip condition are recovered.

Our first step is to transform the given system to a non-dimensionalized form. For this purpose, we introduce the following dimensionless quantities

(9) $$\overset{¯}{x}=\sqrt{\frac{a}{\nu }}x,\;\;\overset{¯}{y}=\sqrt{\frac{a}{\nu }}y,\;\;\overset{¯}{u}=\frac{u}{\sqrt{a\nu }},\;\;\overset{¯}{v}=\frac{v}{\sqrt{a\nu }},\;\;\theta =\frac{T-T_{\infty }}{T_{w}-T_{\infty }}.$$

Insert the scaling (9) into the system described by Eqs (4)–(6) and dropping the bars, the continuity, the momentum and the energy equations become

(10) $$\frac{\partial u}{\partial x}+\frac{\partial v}{\partial y}=0,$$

(11) $$u\frac{\partial u}{\partial x}+v\frac{\partial u}{\partial y}=(1-n)\frac{\partial^{2}u}{\partial y^{2}}+\sqrt{2}n\Gamma a\left(\frac{\partial u}{\partial y}\right)\frac{\partial^{2}u}{\partial y^{2}}-\frac{\sigma B^{2}}{\rho a}u,$$

(12) $$u\frac{\partial \theta }{\partial x}+v\frac{\partial \theta }{\partial y}=\frac{\kappa }{\mu c_{p}}\frac{\partial^{2}\theta }{\partial y^{2}}+\frac{Q_{0}}{\rho c_{p}a}\theta .$$

In the scenario of scaling given in (9), the boundary conditions (7) and (8) take the form

(13) $$u=x+\sqrt{\frac{a}{\nu }}L\frac{\partial u}{\partial y},\;\;v=0,\;\;\theta =1+\sqrt{\frac{a}{\nu }}D_{1}\frac{\partial \theta }{\partial y},\;\;\text{at}\;y=0,$$

(14) u→0,  θ→0  as  y→∞.

Next, to reduce the number of dependent variables and the number of equations, one needs to introduce the stream function $u=\frac{\partial^{\psi }}{\partial y}$, $v=-\frac{\partial \psi }{\partial x}$. The mass-conservation equation Eq. (10) is satisfied automatically and Eqs. (11) and (12) in term of the stream function *ψ*, and take the form

(15) $$\left(\frac{\partial \psi }{\partial y}\frac{\partial \psi }{\partial x\partial y}-\frac{\partial \psi }{\partial x}\frac{\partial^{2}\psi }{\partial y^{2}}\right)=(1-n)\frac{\partial^{3}\psi }{\partial y^{3}}+\sqrt{2}n\Gamma a\left(\frac{\partial^{2}\psi }{\partial y^{2}}\right)\frac{\partial^{3}\psi }{\partial y^{3}}-\frac{\sigma B^{2}}{\rho a}\frac{\partial \psi }{\partial y},$$

(16) $$\left(\frac{\partial \psi }{\partial y}\frac{\partial \theta }{\partial x}-\frac{\partial \psi }{\partial x}\frac{\partial \theta }{\partial y}\right)=\frac{\kappa }{\mu c_{p}}\frac{\partial^{2}\theta }{\partial y^{2}}+\frac{Q_{0}}{\rho c_{p}a}\theta .$$

Again, the induction of the stream function converts the boundary conditions (13) and (14) to

(17) $$\frac{\partial \psi }{\partial y}=x+\sqrt{\frac{a}{\nu }}L\frac{\partial^{2}\psi }{\partial y^{2}},\;\;\frac{\partial \psi }{\partial x}=0,\;\;\theta =1+D_{1}\sqrt{\frac{a}{\nu }}\frac{\partial \theta }{\partial y},\;\;\text{at}\;y=0,$$

(18) $$\frac{\partial \psi }{\partial y}\to 0,\;\;\theta \to 0\;\;\text{as}\;\;y\to \infty .$$

## 3 Analysis

In this section, we perform Lie group analysis to find the new similarity transformations of Eqs (15) and (16). This will reduce the nonlinear partial differential equations to nonlinear ordinary differential equations. For this purpose, we consider the following scaling group of transformation

(19) $$\begin{array}{ccc} \Gamma :x^{⁎} & = & xe^{\varepsilon \gamma_{1}},\;\;y^{⁎}=ye^{\varepsilon \gamma_{2}},\;\;\psi^{⁎}=\psi e^{\varepsilon \gamma_{3}},\\ \theta^{⁎} & = & \theta e^{\varepsilon \gamma_{4}}\;\;,\;\;\Gamma^{⁎}=\Gamma e^{\varepsilon \gamma_{5}}. \end{array}$$

Here *ε* is the parameter of the group Γ and $\gamma_{i}=(i=1,2,\ldots ,5)$ are the arbitrary real number to be determined. The co-ordinates (x,y,ψ,θ,Γ) are transformed to $\left(x^{⁎},y^{⁎},\psi^{⁎},\theta^{⁎},\Gamma^{⁎}\right)$ by the point-transformation (19).

By inserting (19) into Eqs. (15) and (16), we obtain

(20) $$\begin{array}{c} e^{\varepsilon (\gamma_{1}+2\gamma_{2}-2\gamma_{3})}\left(\frac{\partial \psi^{⁎}}{\partial y^{⁎}}\frac{\partial^{2}\psi^{⁎}}{\partial x^{⁎}\partial y^{⁎}}-\frac{\partial \psi^{⁎}}{\partial x^{⁎}}\frac{\partial^{2}\psi^{⁎}}{\partial y^{{⁎}^{2}}}\right)\\ \;=e^{\varepsilon (3\gamma_{2}-\gamma_{3})}(1-n)\frac{\partial^{3}\psi^{⁎}}{\partial y^{{⁎}^{3}}}+e^{\varepsilon (4\gamma_{2}-2\gamma_{3}-\gamma 5)}\left(\sqrt{2}n\Gamma a\left(\frac{\partial \psi^{⁎}}{\partial y^{⁎}}\right)\frac{\partial^{3}\psi^{⁎}}{\partial y^{{⁎}^{3}}}\right)\\ \;-\frac{\sigma B^{2}}{\rho a}e^{\varepsilon (\gamma_{2}-\gamma_{3})}\frac{\partial \psi^{⁎}}{\partial y^{⁎}}, \end{array}$$

(21) $$e^{\varepsilon (\gamma_{1}+\gamma_{2}-\gamma_{3}-\gamma_{4})}\left(\frac{\partial \psi^{⁎}}{\partial y^{⁎}}\frac{\partial \theta^{⁎}}{\partial x^{⁎}}-\frac{\partial \psi^{⁎}}{\partial x^{⁎}}\frac{\partial \theta^{⁎}}{\partial y^{⁎}}\right)=e^{\varepsilon (2\gamma_{2}-\gamma_{4})}\frac{\kappa }{\mu c_{p}}\frac{\partial^{2}\theta^{⁎}}{\partial y^{{⁎}^{2}}}+\frac{Q_{0}}{\rho c_{p}a}e^{-\varepsilon \gamma_{4}\varepsilon }\theta^{⁎}.$$

The transformed system (20) and (21) will remain invariant under the group of transformation Γ, if the exponents in these equations satisfy the following relations

(22) $$\gamma_{1}+2\gamma_{2}-2\gamma_{3}=3\gamma_{2}-\gamma_{3}=4\gamma_{2}-2\gamma_{3}-\gamma 5=\gamma_{2}-\gamma_{3},$$

(23) $$\gamma_{1}+\gamma_{2}-\gamma_{3}-\gamma_{4}=2\gamma_{2}-\gamma_{4}+-\gamma_{4}.$$

From the boundary condition, we have

(24) $$\gamma_{4}=0.$$

We solve Eqs. (22) and (23) simultaneously to obtain

(25) $$\gamma_{1}=\gamma_{1},\;\;\gamma_{2}=0,\;\;\gamma_{3}=\gamma_{1},\;\;\gamma_{4}=0,\;\;\gamma_{5}=-\gamma_{1}.$$

By embedding (25) into the scalings (19), one can observe that the set of transformations is converted into the following one parameter group of transformations

(26) $$\begin{array}{ccc} \Gamma :x^{⋆} & = & xe^{\varepsilon \gamma_{1}},\;\;y^{⋆}=y,\;\;\psi^{⋆}=\psi e^{\varepsilon \gamma_{1}},\\ \theta^{⋆} & = & \theta ,\;\;\Gamma^{⋆}=\Gamma e^{-\varepsilon \gamma_{1}}. \end{array}$$

A Taylor's series expansion of one parameter group of the (26) at ε=0 and retaining term up to the first order in *ε* leads to the following simpler form

(27) $$x^{⋆}-x=x\varepsilon \gamma_{1},\;\;y^{⋆}-y=0,\;\;\psi^{⋆}-\psi =x\varepsilon \gamma_{1},\;\;\theta^{⋆}-\theta =0,\;\;\Gamma^{⋆}-\Gamma =-x\varepsilon \gamma_{1}.$$

From Eq. (27), one can easily deduce that the set of transformation can be written in the form of the following characteristic equations

(28) $$\frac{dx}{x\gamma_{1}}\;\;=\frac{dy}{0}\;\;=\frac{d\psi }{x\gamma_{1}}\;\;=\frac{d\theta }{0}\;\;=\frac{d\Gamma }{-x\gamma_{1}}.$$

We can find the similarity transformations from Eq. (28). From the first two terms in Eq. (28), we have $\frac{dx}{\gamma_{1}x}=\frac{dy}{0}$, which can be integrated to gives

(29) y=constant=η(say).

Equating the first and third terms in Eq. (28) gives $\frac{dx}{x\gamma_{1}}=\frac{d\psi }{\psi \gamma_{1}}$ solving this yield

(30) $$\frac{\psi }{x}=\text{constant}=f(\eta )\;\text{(say)},\text{i.e.},\;\psi =xf(\eta ).$$

Equating the first and fourth term in the Eq. (28) and integrating both sides, we obtain

(31) θ=θ(η).

Similarly, equating the first and the last term of (28) and integrating both sides, we obtain

(32) $$\Gamma =x^{-1}\Gamma_{0}.$$

Thus the new similarity transformation are

(33) $$\eta =y,\;\;\psi =xf(\eta ),\;\;\theta =\theta (\eta ),\;\;\Omega =x\Omega_{0},\;\;\Gamma =x^{-1}\Gamma_{0}.$$

Substituting Eq. (33) into Eqs (15) and (16), we obtain the following nonlinear ordinary differential equations

(34) $$(1-n)f^{\prime \prime \prime }-{\left(f^{\prime }\right)}^{2}+f^{\prime \prime }f+nW_{e}f^{\prime \prime \prime }(f^{\prime \prime })-M^{2}f^{\prime }=0,$$

(35) $$\theta^{\prime \prime }+Pr\left(f0^{\prime }+Q\theta \right)=0,$$

where $W_{e}=\sqrt{2}\Gamma a$, is the Weissenberg number, $M^{2}=\frac{\sigma B^{2}}{\rho a}$ is the Hartmann number, $Q=\frac{Q_{0}}{\rho c_{p}a}$ is the source/sink parameter and $Pr=\frac{\mu c_{p}}{\kappa }$ is the Prandtl number.

Here primes denoted differential with respectively *η*. We solve the system described by Eqs. (34) and (35) subject to the boundary conditions

(36) $$f(0)=0,\;f^{\prime }(0)=1+\alpha f^{\prime \prime }(0),\;\theta =1+b\;\theta^{\prime }(0),$$

(37) $$f^{\prime }\to 0,\;\theta \to 0\;\text{as}\;\eta \to \infty .$$

In the above equations, $\alpha =\sqrt{\frac{a}{\nu }}L$ is the velocity slip parameter and $b=\sqrt{\frac{a}{\nu }}D_{1}$, is the thermal slip parameter, respectively.

The skin friction coefficient $C_{f}$ and the local Nusselt number $Nu_{x}$ is defined

(38) $$C_{f}=\frac{\tau_{w}}{\rho {\left(ax\right)}^{2}},\;\;Nu_{x}=\frac{xq_{w}}{k(T_{w}-T_{\infty })},$$

where the skin friction $\tau_{w}$ and the heat transfer from the plate $q_{w}$ are

(39) $$\tau_{w}=\left(1-n\right)\frac{\partial u}{\partial y}+\frac{n\Gamma }{\sqrt{2}}{\left(\frac{\partial u}{\partial y}\right)}^{2},\;\;q_{w}=-k(\frac{\partial T}{\partial y}).$$

We insert Eqs. (9) and (33) into (38), the dimensionless form of the skin friction and local Nusselt become

(40) $$Re^{\frac{1}{2}}C_{f}=\left[\left(1-n\right)f^{\prime \prime }(0)+\frac{n}{2}We{\left(f^{\prime \prime }(0)\right)}^{2}\right],\;\;Re^{-\frac{1}{2}}Nu_{x}=-\theta^{\prime }(0),$$

where $Re=\frac{ax^{2}}{\nu }$ is the local Reynolds Number.

## 4 Results and discussion

### 4.1 Method of solution

In order to solve the system of nonlinear ordinary differential equations given by (34) and (35) subject to the boundary conditions (36) and (37) are solved numerically by shooting method. The step size Δη=0.001 is used to obtain the numerical solution with $\eta_{max}$, and accuracy to the fifth decimal place is chosen as the criterion of convergence. The numerical computations have been carried out for various values of controlling parameters. The skin friction coefficient and local Nusselt number in the Eq. (40) are presented graphically and discussed in the following section. The present result of skin friction coefficient are compared with those by Akbar et al. [1] are show in Table 1. We notice that the comparisons shows good agreement for each values considered.

**Table 1:** Comparison of skin friction coefficient in the absence of velocity slip parameter *α* = 0 for various values of *M*, *W*_*e*_, *n*.

*n*↓	*M*↓	Akber et al. [1]			Present		
		$W_{e}=0.0$	$W_{e}=0.3$	$W_{e}=0.5$	$W_{e}=0.0$	$W_{e}=0.3$	$W_{e}=0.5$
0.0	0	1	1	1	1	1	1
0.1	0	0.94868	0.94248	0.93826	0.94868	0.94248	0.93826
0.2	0	0.89442	0.88023	0.87026	0.89442	0.88023	0.87026
0.3	0.5	1.02472	0.98804	0.96001	1.02472	0.98804	0.96001
0.3	1.0	1.18322	1.13454	1.09616	1.18322	1.13454	1.09616
0.3	1.5	1.32288	1.26193	1.21235	1.32288	1.26193	1.21235

### 4.2 Discussion

**Figure 1:**
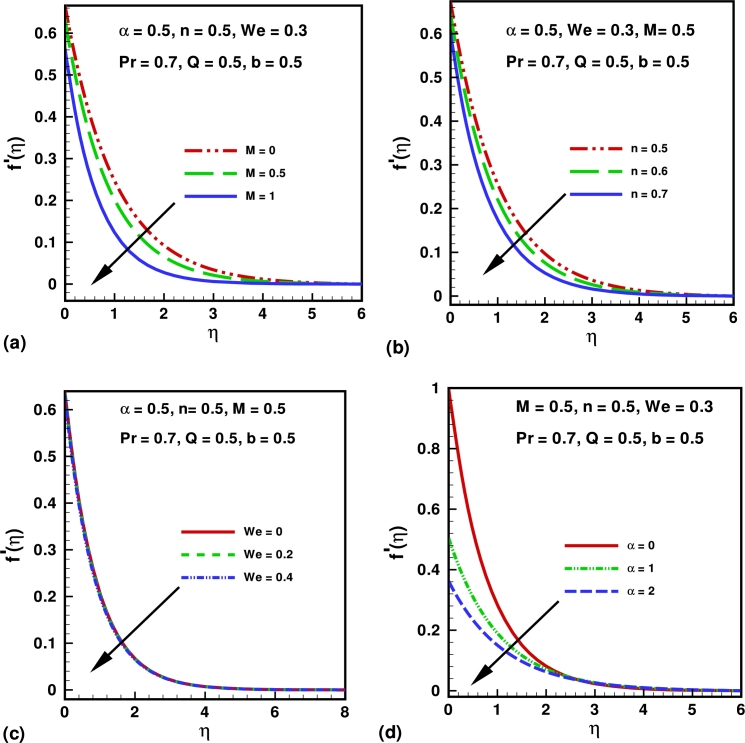
Variation of velocity for different values of (a) Hartmann number (*M*); (b) power law index (*n*); (c) Weissenberg number (*We*) and (d) slip parameter (*α*).

**Figure 2:**
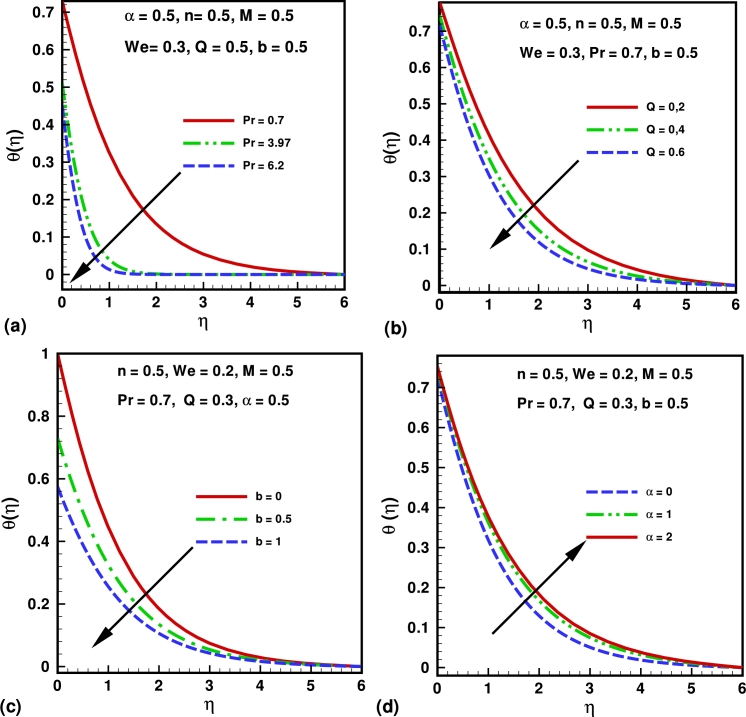
Variation of temperature profile for different values of (a) Prandtl number (*Pr*); (b) source/sink parameter (*Q*); (c) Thermal slip parameter (*b*) and (d) velocity slip parameter (*α*).

**Figure 3:**
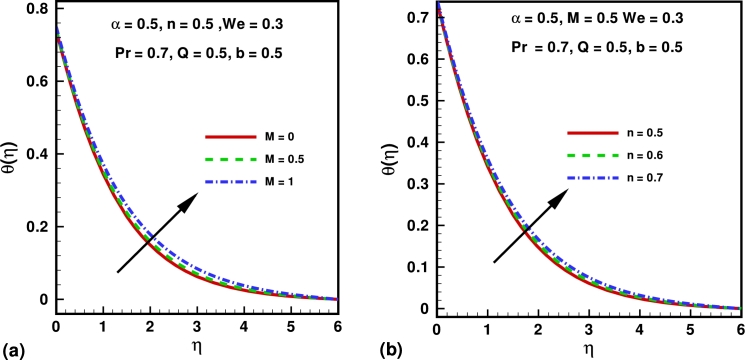
variation of temperature profile for different values of (a) Hartmann number (*M*) and (b) power law index (*n*).

**Figure 4:**
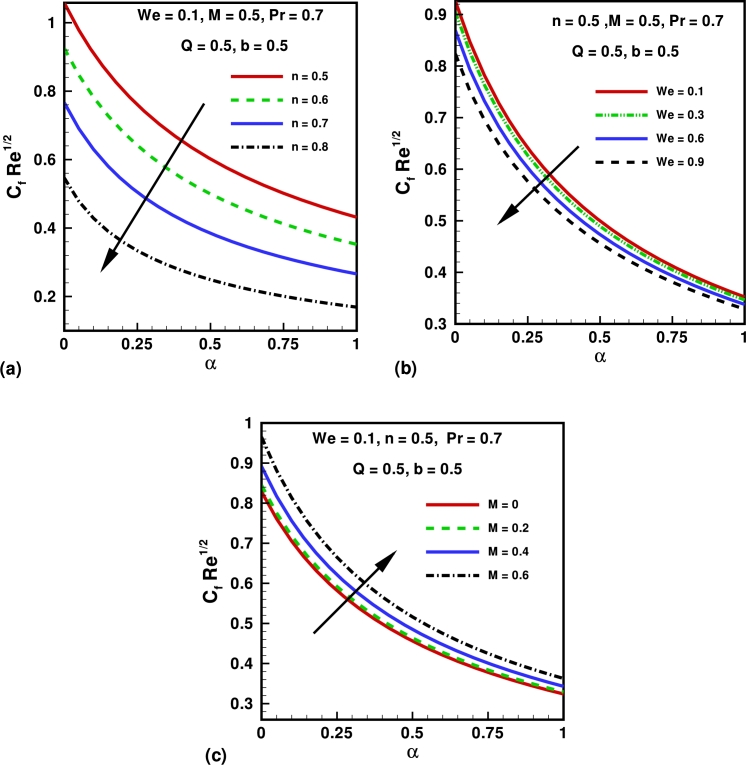
Variation of skin fraction coefficient for different values of (a) power law index (*n*); Weissenberg number (*We*) and Hartmann number (*M*).

**Figure 5:**
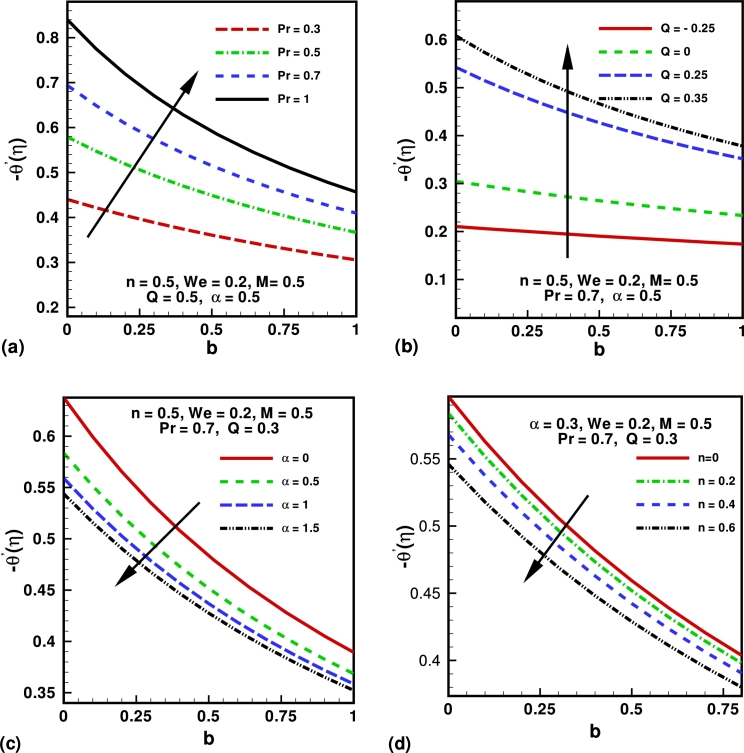
variation of Nusselt numbers for different values of (a) Prandtl number (*Pr*); (b) source/sink parameter (*Q*); (c) velocity slip parameter (*α*) and (d) power law index (*n*).

The numerical solutions are presented through graphs (see Figure 1, Figure 2, Figure 3, Figure 4, Figure 5) for the physical interpretation of the proposed study. These figures describes the influence of Hartmann number *M*, Weissenberg number *We*, power law index *n*, Prandtl number *Pr*, source/sink parameter *Q*, velocity slip parameter *α* and thermal slip parameter *b* on the dimensionless velocity, temperature, skin friction coefficient and local Nusselt number. Figure 1(a) shows the influence of Hartmann number *M* on the velocity profile. Hartmann number is the ratio of electromagnetic force to the viscous force. From the graph it is clear that by increasing the values of *M*, the velocity profile decrease and boundary layer thickness also deceases. This is happening due to increasing value of *M* which tends to increase the Lorentz force, and produces more resistance to the transport phenomena. Figure 1(b) shows the influence of the power law index *n* on the velocity profile of the flow. It is observed that by increasing the valves of *n* both boundary layer thickness and the velocity profile decreases. Figure 1(b) depicts that the behavior of law index *n* on the velocity profile is qualitatively same in Figure 1(a). Figure 1(c) shows the effects of Weissenberg number *We* over the velocity function $f^{\prime }(\eta )$. It is clear that velocity profile decreases by increasing the values of *We*, because after increasing Weissenberg number *We* the relaxation time increase which offers more resistance to flow. Consequently, the boundary layer thickness decreases with an increase in *We*. Figure 1(d) show the influence of slip parameter *α* on the velocity profile. From the graph it is clear that the velocity profile decreases by increasing the values of *α*. Also velocities boundary layer thicknesses is a decreasing functions of *α*. Figure 2(a) describes the impact of the Prandtl number *Pr* over the temperature profile. It can be easily deduced form the figure that the temperature and the thickness of the thermal boundary layer decrease with in increase in *Pr*, or equivalently, in the fluid thermal conductivity. Figure 2(b) describes the impact of the skin parameter *Q* over the temperature profile. It is clear from the figure that temperature profile decreases by increasing the values *Q*. This shows that an increase in the sink parameter reduces the thickness of the thermal boundary layer. Figure 2(c) describes the impact of the thermal slip parameter *b* on the temperature profile. It is clear from the figure that temperature profile decreases strictly with the an increase in the thermal slip parameter. We also note that the momentum equation is independent of the thermal slip parameter. Figure 2(d) describes the impact of the velocity slip parameter *α* over the temperature profile. From the graph it is clear that the temperature profile increases by increasing the values of *α*. Figure 3(a) shows the influence of Hartmann number on the temperature profile. From the figure graph it is clear that The temperature profile increases as *M* increases. Also thermal boundary layer thicknesses is a increasing functions of *M*. Figure 3(b) shows the influence of the power law index *n* on the temperature profile. From Figure, it is observed that for higher values of *n*, it increases both boundary layer thickness and the magnitude of temperature profile.

Effect of physical parameter on the skin friction coefficients and local Nusselt number are presented in Figure 4, Figure 5. Figure 4(a) represents the profiles of the skin friction versus the velocity slip parameter *α* for serval values of the power law index *n*. It can be easily deduced that for a fixed value of *α*, the skin friction decrease as we increase the value of the power law index *n*. Also the skin friction decreases with increasing *α*. From Figure 4(b) it can be easily deduced that for the fixed value of *α*, the skin friction decrease as we increase the value of Weissenberg number *We*. In Figure 4(c) it depicts that for higher values of Hartman number *M*, skin friction coefficient presents the increasing behavior corresponding to the increasing values of *α*. Excellent agreement is presented for skin friction coefficient shown in Table 1 in the absence of velocity slip parameter *α* [1]. Figure 5 shows the influence of governing parameters on dimensionless heat transfer rates. Figure 5(a) depicts that for higher values of Prandtl number *Pr* local Nusselt number present the increasing behavior corresponding to the increasing values thermal slip parameter *b*. Figure 5(b) shows the influence of source or skin *Q* and thermal slip parameter *b* on local Nusselt number. It is observed form the figure that local Nusselt number increase as skin parameter *Q* increase and decreases with increasing thermal slip parameter *b*. Figure 5(c) show, the influence velocity parameter *α* and thermal slip parameter *b* on local Nusselt number. It is observed form the given figures that local Nusselt number decrease as both the parameters *a* and *b* increases. Figure 5(d) shows the influence of power law index *n* and thermal slip parameter *b* on local Nusselt number. It is observed form the figure that local Nusselt number decrease as both the parameters power law index *n* and thermal slip parameter *b* increases.

## 5 Conclusions

We investigated the MHD flow of tangent hyperbolic fluid flow and heat transfer towards a stretching sheet with velocity and thermal slip. The governing non-liner partial differ equation are reduced into a system of coupled non-linear ordinary differential equations using similarity transformation developed by lie group analysis. The main results of the present analysis can be listed below.

Behavior of Hartmann number *M* and power law index *n* are qualitatively similar on the velocity profile. The velocity profile $f^{\prime }(\eta )$ decreases with increasing values of velocity slip parameter *α* and the boundary layer thickness also decreases. The dimensionless temperature θ(η) increases with increasing velocity slip parameter *α*, Hartmann number *M*, power law index *n* and decreases with increasing Prandtl number *Pr*, source/sink parameter *Q* and thermal slip parameter *b*. The skin friction coefficient increases with Hartmann number *M* and decreases with power law index *n*, Weissenberg number *We* and velocity slip parameter *α*. The Prandtl number *Pr* and source/sink parameter *Q* has increasing effects on local Nusselt number and it decreases with velocity slip parameter *α* and thermal slip parameter *b*. Present results shows good agreement with the represent result [1].

## Declarations

### Author contribution statement

Zakir Ullah: Conceived and designed the experiments; Performed the experiments; Analyzed and interpreted the data; Wrote the paper.

Gul Zaman: Contributed reagents, materials, analysis tools or data; Wrote the paper.

### Funding statement

This research did not receive any specific grant from funding agencies in the public, commercial, or not-for-profit sectors.

### Competing interest statement

The authors declare no conflict of interest.

### Additional information

No additional information is available for this paper.
